# Behavioural sensitivity to binaural spatial cues in ferrets: evidence for plasticity in the duplex theory of sound localization

**DOI:** 10.1111/ejn.12402

**Published:** 2013-10-28

**Authors:** Peter Keating, Fernando R Nodal, Andrew J King

**Affiliations:** Department of Physiology, Anatomy and Genetics, University of OxfordParks Road, Oxford, OX1 3PT, UK

**Keywords:** auditory localization, interaural level difference, interaural time difference, phase ambiguity, spatial hearing, training

## Abstract

For over a century, the duplex theory has guided our understanding of human sound localization in the horizontal plane. According to this theory, the auditory system uses interaural time differences (ITDs) and interaural level differences (ILDs) to localize low-frequency and high-frequency sounds, respectively. Whilst this theory successfully accounts for the localization of tones by humans, some species show very different behaviour. Ferrets are widely used for studying both clinical and fundamental aspects of spatial hearing, but it is not known whether the duplex theory applies to this species or, if so, to what extent the frequency range over which each binaural cue is used depends on acoustical or neurophysiological factors. To address these issues, we trained ferrets to lateralize tones presented over earphones and found that the frequency dependence of ITD and ILD sensitivity broadly paralleled that observed in humans. Compared with humans, however, the transition between ITD and ILD sensitivity was shifted toward higher frequencies. We found that the frequency dependence of ITD sensitivity in ferrets can partially be accounted for by acoustical factors, although neurophysiological mechanisms are also likely to be involved. Moreover, we show that binaural cue sensitivity can be shaped by experience, as training ferrets on a 1-kHz ILD task resulted in significant improvements in thresholds that were specific to the trained cue and frequency. Our results provide new insights into the factors limiting the use of different sound localization cues and highlight the importance of sensory experience in shaping the underlying neural mechanisms.

## Introduction

Although first proposed more than a century ago (Strutt, 1907), the ‘duplex theory’ of sound localization provides a remarkably successful account of the way in which humans localize pure tones (Blauert, 1997; Macpherson & Middlebrooks, 2002). According to this theory, spatial hearing in the horizontal plane transitions between two distinct mechanisms as the frequency of a sound is increased. Whereas low-frequency sounds are localized using interaural time differences (ITDs), which are produced by differences in path length between the sound source and each ear, high-frequency sounds are localized primarily on the basis of interaural level differences (ILDs), which are created by a combination of the directional filtering properties of the external ears and the acoustical shadowing effect of the head. However, whilst the duplex theory can account for spatial processing of pure tones in some non-human species (Wakeford & Robinson, 1974; Brown *et al*., 1978; Houben & Gourevitch, 1979), there are key exceptions (Heffner & Heffner, 2003; Takahashi, 2010; Wesolek *et al*., 2010). In species where the duplex theory does apply, it is also of interest to determine whether the range of sound frequencies over which sounds are lateralized using ITDs and ILDs is fixed or whether this can be modified by sensory experience. The answer to this question is important not only because it would provide insight into the mechanisms underlying behavioural sensitivity to binaural spatial cues, but also because it has important implications for plasticity in the adult brain as well as rehabilitation strategies following hearing loss.

Over the past few decades, the ferret has been used extensively for studying sensory processing and plasticity, and is particularly suitable for investigating fundamental aspects of spatial hearing (reviewed by King *et al*., 2011). Although there have been many behavioural studies of spatial hearing in this species, these studies have tended to focus on sound localization in the free field (Kavanagh & Kelly, 1987; King & Parsons, 1999; Parsons *et al*., 1999; Kacelnik *et al*., 2006; Bizley *et al*., 2007; Nodal *et al*., 2010a; Irving *et al*., 2011). Because spatial cues typically co-vary with one another under free-field conditions (Blauert, 1997), it is difficult to manipulate individual cues in isolation, thereby preventing the duplex theory from being tested directly. More recently, methods have been developed that enable sounds to be presented to ferrets over earphones while they perform a behavioural task (Nodal *et al*., 2010b). Using this technique to present broadband noise stimuli, we have shown that the ITD and ILD sensitivity of ferrets is comparable to that of humans (Keating *et al*., 2013b). It is not presently known, however, over what frequency range each of these cues operates in ferrets, or the extent to which acoustical or neural factors account for behavioural sensitivity at different sound frequencies.

In this study, we presented pure tones to adult ferrets over earphones and measured their ITD and ILD sensitivity as a function of frequency. Overall, we found that the duplex theory can successfully account for the lateralization of tones in this species and show that the underlying mechanisms can be altered in a frequency- and cue-specific way by behavioural training.

## Materials and methods

Four adult (1–3 years old) pigmented female ferrets from our breeding colony were used for the purposes of this study. All procedures were performed under licences granted by the UK Home Office and met with ethical standards approved by the University of Oxford.

### Behavioural apparatus

**Fig. 1:**
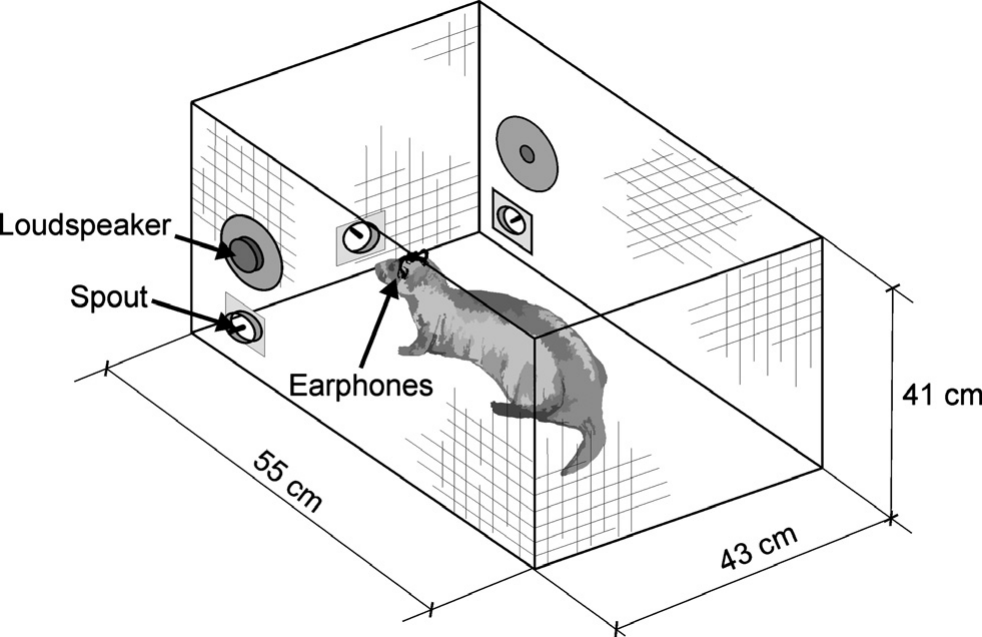
Schematic showing experimental apparatus used for behavioural testing. Free-field stimuli were presented from the two loudspeakers during initial training, prior to presenting stimuli via a closed-field sound delivery system comprising earphones positioned in head-mounted holders.

Details of the setup used for behavioural testing have been described previously (Keating *et al*., 2013b). Briefly, these measurements were performed in a standard mesh cage with a solid plastic floor, which was enclosed by a sound-insulated box lined with acoustic foam (MelaTech; Hodgson & Hodgson Ltd, Melton Mowbray, UK). The animal initiated a trial by inserting its nose into a poke-hole located in the middle of the front wall of the cage. After a variable delay, a sound was presented and the animal responded by poking its nose into poke-holes located on either side of the testing cage (Fig. 1). Correct responses were rewarded with a specified amount of water (150–300 μL per trial) that was delivered via spouts situated in each of the poke-holes. Incorrect responses were followed by correction trials on which the same stimulus was presented. Stimuli were generated in Matlab (The Mathworks, Natick, MA, USA), sent to a real-time processor (RP2; Tucker-Davis Technologies, Alachua, FL, USA), amplified and presented to the animal either via loudspeakers located on either side of the testing cage (FRS 8; Visaton, Crewe, UK) or via earphones (RP-HV280; Panasonic, Bracknell, UK).

### Closed-field sound delivery system

Earphones were connected to titanium earphone holders that were, in turn, attached to the skull via a cranial implant. The design of both the implant and the earphone holders, as well as the surgical details involved in implantation, have been detailed elsewhere (Nodal *et al*., 2010b) and so are described only briefly here. The earphone holders were designed so that they could be easily disconnected and reattached to the implant, and were removed whenever the animal was not performing the task. They were also individually adjustable, enabling them to be consistently positioned immediately in front of the ear canals for each animal.

The cranial implant comprised two bolts encased in bone cement (CMW1 Bone Cement; DePuy CMW, Lancashire, UK), which was attached directly to the skull using dental adhesive (Super-bond C&B; Sun Medical Co, Shiga, Japan). Prior to implantation under sterile conditions, animals were anaesthetized with a combination of medetomidine hydrochloride (0.022 mg/kg i.m.; Domitor; Pfizer Ltd, Sandwich, UK) and ketamine (5 mg/kg; Ketaset; Fort Dodge Animal Health, Southampton, UK). The left radial vein was then cannulated and the animal was intubated and mechanically ventilated, thereby allowing the anaesthetic agent to be switched to 0.5–1.5% isoflurane (IsoFlo; Abbott Laboratories Ltd, Kent, UK). This was followed by i.m. administration of atipamezole (Antisedan; Pfizer) to reverse the effects of medetomidine, atropine (0.006 mg/kg; Atrocare; Animalcare Ltd, York, UK) to reduce secretions, and buprenorphine (0.03 mg/kg; Vetergesic; Alstoe Animal Health, Melton Mowbray, UK) and meloxicam (0.2 mg/kg; Metacam; Boehringer Ingelheim, Terrassa, Spain) to provide perisurgical analgesia. Local anaesthetic (Elma; Astra Zeneca Luton, UK) was applied to stereotaxic pressure points and carbomer (Viscotears; Lewis Pharmaceuticals Ltd, Doncaster, UK) was applied to the eyes. Electrocardiogram, end-tidal CO_2_ and core body temperature were monitored throughout.

Once the head was positioned in a stereotaxic frame, a midline incision was used to expose the dorsal part of the skull and the temporal muscles were displaced laterally. After cleaning the skull with 1% citric acid, the implant was progressively built up using layers of bone cement. Once the implant was complete, the skin and temporal muscles were repositioned and sutured together both to the front and rear of the implant. Following recovery from anaesthesia, the animal was given buprenorphine for 3 days and meloxicam for 5 days.

### Behavioural testing

Prior to attachment of the cranial implant, animals were trained to perform a two-alternative forced-choice task using free-field loudspeakers located on either side of the testing cage (Fig. 1). During the initial training, the animal had to insert its nose into the centre spout poke-hole for 200 ms, after which a sound was played from one of the loudspeakers. Animals were initially trained using broadband stimuli and these stimuli were used exclusively until animals were familiar with the procedural aspects of the task. In particular, tones were only introduced once animals progressed to performance of the ILD and ITD tasks.

Once the animals had learned the mechanics of this task and were performing ∼ 100 trials on each session, we gradually increased the delay at the centre spout prior to trial initiation and allowed it to vary from 1 to 7 s. Identical stimuli were then presented over both loudspeakers simultaneously, but with a 30-dB difference in level between them, with the animals required to judge the perceived location of the sound by licking the response spout on the side of the more intense sound. To avoid a bias towards one speaker location, an incorrect response was followed by correction trials (same stimulus and location) until the animals responded correctly. Once the animals attained a stable performance of > 97% correct for several days, they were judged trained and ready for implantation of the cranial support. In total, this period of training took 1–3 weeks depending on the animal.

After implantation, animals were again tested to confirm that they could still discriminate between the same free-field noise bursts. Earphones were then attached to enable closed-field stimuli to be presented with the same ILD of 30 dB. After again achieving a performance level of > 97% correct over several days, the ILD or ITD between the two earphones was varied so that psychometric functions could be obtained for pure tones of different frequencies.

When training animals on the procedural aspects of the task, we wanted to minimize any perceptual learning that might occur during this process. For this reason, we presented animals with very large cue values that were easy to distinguish between (Keating *et al*., 2013b). In the case of ILDs, this can be readily achieved by increasing the magnitude of the cue. Very large ITDs, however, are not necessarily easy to lateralize due to phase ambiguities. The spatial percept of ITDs also breaks down when these cues are made too large, with the acoustical input to each ear separating into two distinct sounds separated in time. Consistent with previous work (Keating *et al*., 2013b), we therefore elected to use large ILD cues alone during preliminary training. Although this procedure initially familiarized the animals with the ILD task only, they were able to transfer immediately to the ITD task. Furthermore, we have previously shown using the same approach that ITD thresholds obtained for broadband noise during the first few sessions are very similar to those measured after extensive training on an ITD task (Keating *et al*., 2013b). This means that the protocol adopted here did not confer a selective advantage on performance in the ILD task, but rather enabled animals to lateralize accurately on the basis of either cue.

Animals were required to perform both an ILD task and an ITD task in separate blocks. For the ITD task, the ILD was set to 0 and ITDs were allowed to vary randomly between a set of predefined values (–80 to 80 μs in increments of 20 μs), where negative values denote stimuli that favoured the left ear. Measurements of ILD sensitivity were performed in two different ways, with the ITD set to 0 in each case. For the majority of measurements, the ILD was varied randomly between –5 and 5 dB (specifically –5, –3, –1.5, –0.5, 0, 0.5, 1.5, 3 and 5 dB), in a manner directly analogous to that used to assess ITD sensitivity. In situations where thresholds could not be estimated reliably, cue values were scaled by a factor from 2 to 4 to assess whether thresholds could be estimated more reliably using larger values. In the case of high-frequency tones, we also restricted the ITD range to ± 60 μs for some sessions to see whether this would improve task performance through a reduction in phase ambiguity.

In the case of ILDs, animals were initially tested with the highest frequencies used before moving on to stimuli with a lower frequency. Similarly, when assessing the frequency dependence of ITD sensitivity for frequencies below 1 kHz, animals were initially tested on 1-kHz stimuli before moving on to stimuli with a lower frequency. Conversely, when measuring the frequency dependence of ITD sensitivity at frequencies above 1 kHz, animals were initially tested using 1-kHz stimuli before progressing to stimuli with a higher frequency. The order in which ITDs or ILDs were presented was varied across different subjects; no obvious effects of testing order were apparent.

To investigate the effects of training on the lateralization of 1-kHz tones, however, we switched to an adaptive technique (Levitt, 1971) in which the ILDs presented were determined by the responses of each subject. Two staircases were randomly interleaved and used to target different points on the psychometric function, each of which was a variant of a two-down, one-up procedure. The first of these staircases used an initial ILD value of 15.5 dB. The ILD was then shifted toward the left (i.e. became more negative) following two consecutive responses made to the right, and was shifted toward the right following a single response to the left. This ensured that this staircase converged on ILD values that elicited responses to the right on 70.7% of trials. For the second staircase, the logical contingencies were reversed, thereby allowing the staircase to converge on ILD values that elicited responses to the right on 29.3% of trials. The step size for each staircase was initially set to 8 dB and was halved each time there was a reversal in the staircase until it reached a minimum of 1 dB. This method minimized the number of trials that were either too difficult or too easy for the animal, concentrating on ILD values that were lateralizable but perceptually challenging. In this way, we aimed to maximize any training-induced improvements in task performance.

The experiment to investigate the effects of training using a 1-kHz tone on ILD thresholds was carried out after all other testing had taken place. Throughout this training period, subjects were occasionally tested on either a 2-kHz ILD task or a 1-kHz ITD task to assess whether any improvements transferred either across frequency or localization cue, respectively.

### Stimuli

In all sessions, animals were required to lateralize 200-ms pure tones of varying frequencies (specifically 250, 500, 1000, 2000, 3000 and 4000 Hz). In the ITD task, all stimuli were presented at 65 dB sound pressure level (SPL). ITDs were created by introducing an interaural phase difference (IPD) between the tones presented to each ear. To minimize ITD information available in the stimulus onset and offset, a 50-ms cosine ramp was added to the beginning and end of each stimulus. These ramps were simultaneously gated in each ear, ensuring that useful ITD information was available only in the fine structure of the tone and not its envelope. Within a single session, all ITD stimuli were presented at the same level. ILDs were imposed by either increasing or decreasing the levels in each ear in such a way that the average binaural level (ABL) was not altered. To ensure that animals could not perform the task using monaural level cues in the ILD task, the ABL was allowed to vary randomly from 60 to 70 dB SPL across trials.

### Estimating the maximum unambiguous ITD

Although the physiological ITD range can be estimated from the radius of the head under the assumption of a spherical head model, more accurate estimates can be obtained through direct acoustical measurements (Schnupp *et al*., 2003). Whilst these measurements are unavailable for many species, the typical physiological ITD range has been experimentally determined to be approximately ± 646 μs for humans (Algazi *et al*., 2001), ± 400 μs for macaques (Spezio *et al*., 2000; Scott *et al*., 2009) and ± 210 μs for ferrets (Schnupp *et al*., 2003). The analyses reported here therefore used these estimates for all calculations.

The maximum unambiguous ITD (ITD_MaxUnambig_), i.e. the largest value where, for a given frequency, the IPD corresponds to a single ITD, was initially determined using numerical simulation, but can be calculated analytically using the physiological ITD limit (ITD_Lim_) and frequency (*f*) as follows:


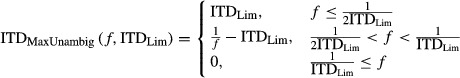


where ITD_MaxUnambig_ has a lower bound of 0 as it represents a magnitude, and with an upper bound determined by the fact that the maximum ITD experienced is equal to ITD_Lim_. This equation accurately captured the structured boundary between ambiguous and unambiguous frequency–ITD combinations, as determined numerically.

### Data analysis

To estimate measures of threshold and bias, psychometric functions were constructed by plotting the percentage of trials on which an animal responded to the right as a function of either ITD or ILD. Although the number of trials varied across sessions, animals typically completed ∼ 100 trials in each. Sessions in which fewer than 50 trials were completed were excluded from analysis. Probit analysis was then used to fit sigmoidal curves to these data, which enabled thresholds to be determined for each session. Because subjects were tested using the same task and stimulus in multiple sessions, statistical tests of significance were generally conducted on thresholds obtained from individual sessions using mixed-effects analyses of variance (anovas) with subject as a random factor. *Post-hoc* analyses were then performed using Tukey's honestly significant difference (HSD) test to correct for multiple comparisons.

## Results

### Frequency dependence of ITD sensitivity

**Fig. 2:**
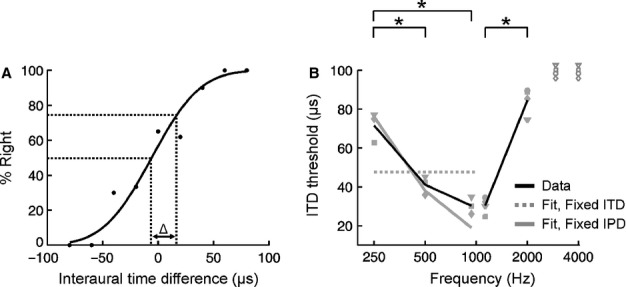
Effect of sound frequency on sensitivity to interaural time differences (ITDs). (A) Psychometric function for an individual session using 1-kHz tones showing the percentage of trials on which a subject responded to the right as a function of ITD. Black markers indicate raw data, with the results of a probit fit shown by a solid black line. Threshold (Δ) is defined as the difference between ITD values that are associated with responses to the right on 50 and 75% of trials. Dotted black lines illustrate the derivation of these values using the probit fit. (B) ITD thresholds are plotted for pure tones of different frequency. Filled grey markers show thresholds for individual subjects, with the mean across subjects indicated in black. Data were obtained at 1 kHz prior to measuring ITD sensitivity at low frequencies (offset slightly to the left) and again prior to determining the upper frequency limit of ITD sensitivity (offset slightly to the right). At lower frequencies, grey lines show the best fit to the averaged data under the assumption of fixed sensitivity to either ITDs (dotted) or IPDs (solid). Open symbols (at 3 and 4 kHz) indicate that thresholds could not be obtained. Asterisks denote significant differences (*P* < 0.05, corrected for multiple comparisons).

In humans, ITD sensitivity is greatest at approximately 1 kHz and declines at frequencies that are either higher or lower than this (Zwislocki & Feldman, 1956; Brughera *et al*., 2013). We therefore wanted to assess whether ITD sensitivity in the ferret shows a similar dependence on sound frequency. To do this, we used customized earphones to present ferrets with pure tones at frequencies between 250 Hz and 4 kHz and varied the ITD across trials. Stimuli were created in such a way that no useful ITD information was available either in the stimulus onset or offset, thereby forcing subjects to rely upon ITDs contained in the ongoing portion of the stimulus. Subjects were then required to lateralize these stimuli correctly to receive a small fluid reward. For each ITD value, we measured the proportion of trials on which the animal responded to the right and used these data to construct psychometric functions (Fig. 2A). Probit analysis (Finney, 1971) was then used to fit sigmoidal curves to the data, and thresholds were measured by calculating the difference between ITD values that were associated with responses made to the right on 50 and 75% of trials (Fig. 2A). This definition is consistent with that used in previous lateralization studies in ferrets (Keating *et al*., 2013b) and other species (Zwislocki & Feldman, 1956; Klumpp & Eady, 1957; Wakeford & Robinson, 1974; Yost, 1974; Scott *et al*., 2007) and means that smaller thresholds were obtained by subjects that were more sensitive to changes in ITD. Because different mechanisms are thought to be responsible for the decline in ITD sensitivity at frequencies above and below 1 kHz (Brughera *et al*., 2013), we first consider the frequency dependence of ITD sensitivity below 1 kHz before turning our attention to ITD sensitivity above 1 kHz.

For pure tones with a frequency of 1 kHz, ITD thresholds were ∼30 μs (Fig. 2B). At frequencies below 1 kHz, however, thresholds increased substantially (*F*_2,25_ = 33.0, *P* < 0.0001, mixed-effects anova), with the thresholds obtained using 250-Hz tones being significantly higher than those obtained at other frequencies (*P* < 0.05, *post-hoc* test; Fig. 2B). When an ITD is experienced for the ongoing portion of a pure tone stimulus, it is thought that the auditory system does not have direct access to the ITD itself. Instead, the auditory system estimates an IPD and infers the ITD from this estimate (Schnupp *et al*., 2011). Over a similar frequency range (0.25–1 kHz), previous work has shown that measurements of ITD sensitivity (Fitzpatrick & Kuwada, 2001) are consistent with a model that assumes an IPD threshold that is constant as a function of sound frequency (Houben & Gourevitch, 1979; Scott *et al*., 2007). Because tones of different frequencies differ in their period, a particular phase delay will translate to a different time delay at each frequency. This means that a fixed IPD will equate to a different ITD at each possible frequency. Using the same data as before, we therefore fitted the data using a routine that was constrained so that the IPD threshold was constant with respect to frequency. This fit (Fig. 2B, continuous grey line) approximated our data much more closely than a fit based on the assumption that ITD sensitivity is fixed with respect to frequency (Fig. 2B, dashed line), producing a significantly smaller root mean square error (*P* = 0.03; bootstrap test). Our data are therefore more consistent with a neural mechanism that is sensitive to a fixed IPD rather than a fixed ITD, although the thresholds measured actually lay between these two possibilities.

**Fig. 3:**
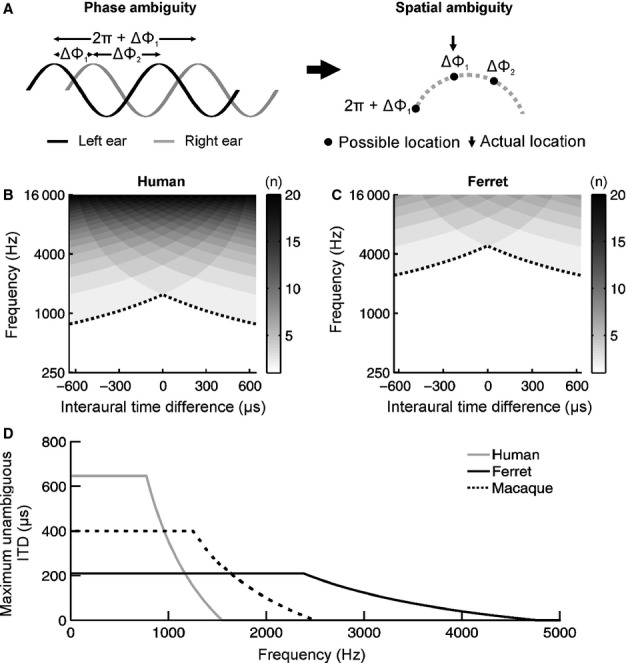
Phase ambiguity is produced by an interaction between ITD magnitude and sound frequency. (A) Phase ambiguity occurs because it is unclear whether the waveform in the right ear (grey) is delayed (ΔΦ_1_) or advanced (ΔΦ_2_) with respect to the waveform in the left ear (black). Assuming that the waveform is delayed in the right ear, it is also difficult to distinguish between a particular IPD (ΔΦ_1_) and other IPDs that differ either by a full period of the waveform (ΔΦ_1_ + 2π) or multiples thereof (i.e. ΔΦ_1_ + *n*2π). Spatial ambiguity can therefore occur whenever the IPD is consistent with more than one ITD in the physiological range. (B, C) For each combination of sound frequency and ITD, we determined the number of physiologically plausible ITDs corresponding to a single IPD (i.e. we measured the degree of spatial ambiguity). Where this value is equal to one (white region), this means that a particular combination of sound frequency and ITD is unambiguous. Values > 1 (grey) denote combinations of frequency and ITD that produce spatial ambiguity, with higher values (darker shades) indicating greater ambiguity with respect to the actual ITD. The black dotted lines depict the boundary between spatially unambiguous (below the line) and ambiguous (above the line) frequency–ITD combinations. The larger physiological ITD range experienced by humans (B) is expected to produce spatial ambiguity at lower frequencies than in ferrets (C), which have much smaller heads and therefore experience a much smaller ITD range. The curve of the dotted lines also indicates that, as frequency is increased, spatial ambiguity should initially occur for large ITDs produced by peripherally located sound sources, and then spread to more central locations (close to 0) as the sound frequency is increased further. (D) The maximum spatially unambiguous ITD is plotted for species of differing head size as a function of sound frequency. Pure tones with a frequency of 2 kHz are spatially ambiguous for humans, but not ferrets. Data are also shown for macaque monkeys (based on Spezio *et al*., 2000; Scott *et al*., 2009), where 2-kHz tones are spatially unambiguous only for ITDs < ∼ 100 μs, corresponding to locations close to the midline.

Whilst fixed IPD sensitivity predicts progressively lower ITD thresholds at higher sound frequencies, the neural representation of IPD is vulnerable to failures in phase locking in the auditory nerve, which plays an important role in conveying information about the temporal structure of the stimulus. Although some species show phase-locked responses across the full range of hearing (Köppl, 1997), phase locking in the auditory nerve of mammals, including ferrets (Sumner & Palmer, 2012), fails at higher frequencies, which would be expected to produce a decline in ITD sensitivity. Lateralization can also become problematic at high frequencies because ITDs become spatially ambiguous (Saberi *et al*., 1998). This happens in situations where the same IPD is caused by more than one ITD within the physiological ITD range, which is determined by the path length between the two ears. In the case of narrowband sounds, this can occur because it is often unclear whether the waveform in one ear is advanced or delayed with respect to the waveform in the other ear. Even when it is clear that the waveform arrives first in one ear, it can be difficult to distinguish a particular ITD from other ITDs that differ either by a full period of the waveform or multiples thereof (Fig. 3A). In this way, a given IPD may be consistent with many different ITDs. At sufficiently low frequencies, each ITD is uniquely identifiable from its corresponding IPD as any phase equivalent values will lie outside the physiological ITD range and will therefore not be encountered naturally. At higher frequencies, however, a given IPD will correspond to a number of naturally occurring ITDs, resulting in spatial ambiguity (Fig. 3A).

To determine the spatial ambiguity that would be expected for different ITDs as a function of frequency, we simulated the general process that is thought to occur within the auditory system. In particular, for each possible combination of frequency and ITD (sampled at a resolution of 1 Hz and 1 μs, respectively), we converted the ITD into an equivalent IPD and then tried to infer what the ITD was on the basis of IPD alone. To do this, we determined the number of physiologically plausible ITDs that produce the same IPD and therefore potentially the same spatial percept. In other words, we measured the degree of spatial ambiguity for different combinations of frequency and ITD. A single physiologically plausible ITD meant that the ITD was unambiguous, whereas more than one value signified spatially ambiguous ITDs. As the number of ITDs corresponding to a single, frequency-independent IPD depends on the physiological ITD range, which can differ dramatically across species, we performed this analysis separately for a typical human (Fig. 3B) as well as a typical ferret (Fig. 3C).

In comparison with humans (Fig. 3B), in the ferret (Fig. 3C), ITD ambiguity is both shifted toward higher frequencies and much less pronounced. In both species, however, a number of points were clear. First, whilst phase ambiguities become inescapable at sufficiently high frequencies (typically frequencies that have a wavelength shorter than the path length between the two ears), they are completely absent at sufficiently low frequencies. Second, the boundary between frequency–ITD combinations that are unambiguous and those that are ambiguous (Fig. 3B and C, dotted lines) depends on the ITD. For example, in humans, for a frequency of 1000 Hz, there is only one physiologically plausible ITD close to the midline (i.e. count equal to 1, as denoted by the white region), whereas two ITD values within the physiological range will be generated at more peripheral locations (light grey regions) (Fig. 3B). Thus, ITDs become ambiguous as they deviate from the midline, but the precise point at which ambiguity occurs depends on the sound frequency. In other words, the maximum unambiguous ITD is frequency-dependent. To capture this intuition analytically, we therefore derived an expression for the maximum unambiguous ITD (see Materials and methods) and plotted the results for different species in Fig. 3D.

If the upper frequency limit of tonal ITD sensitivity were determined by phase ambiguity, Fig. 3D predicts that ferrets should be able to lateralize tones that exceed 3 kHz, a higher value than that found in species, such as humans or macaque monkeys, which have larger heads. On the other hand, if tonal ITD sensitivity declines at frequencies < 3 kHz, then this would be more consistent with a failure in phase locking. To distinguish between these two possibilities, we measured pure tone ITD thresholds for ferrets using frequencies ≥ 1 kHz (Fig. 2B). As expected, ferrets were able to lateralize 2-kHz tones on the basis of ITDs, something that humans are unable to do (Zwislocki & Feldman, 1956; Brughera *et al*., 2013). Importantly, however, we found that ITD thresholds were significantly higher at 2 kHz than at 1 kHz (*F*_1,56_ = 124.2, *P* < 0.0001, mixed-effects anova; Fig. 2B). We were also unable to obtain ITD thresholds at frequencies ≥ 3 kHz, with ferrets completely unable to perform the ITD task under such conditions. These results therefore suggest that the upper frequency limit of pure tone ITD sensitivity in this species is not a consequence of phase ambiguity, but is instead more likely due to the neurophysiological characteristics of its auditory system.

### Frequency-dependence of ILD sensitivity

**Fig. 4:**
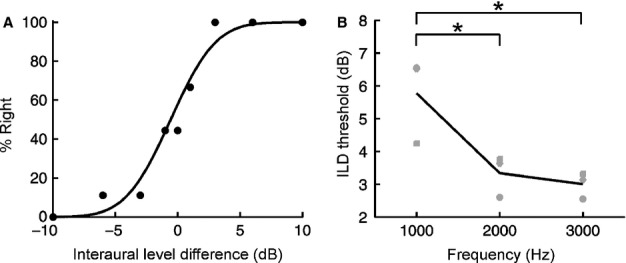
Effect of sound frequency on interaural level difference (ILD) sensitivity. (A) Psychometric function for an individual session using 3-kHz tones showing the percentage of trials on which the animal responded to the right as a function of ILD. (B) ILD thresholds plotted as a function of frequency. Grey markers show data for individual animals, with the black line showing mean thresholds across subjects. Asterisks denote significant differences (*P* < 0.05, corrected for multiple comparisons).

To investigate the frequency dependence of ILD sensitivity in the ferret, we varied the ILD of pure tones, constructed psychometric functions and computed thresholds as before (Fig. 4A and B). In contrast to previous work (Mills, 1960; Wakeford & Robinson, 1974; Scott *et al*., 2007), we found that there was a very strong effect of sound frequency on ILD sensitivity (*F*_2,25_ = 12.41, *P* = 0.0002; mixed-effects anova). At 3 kHz, thresholds were ∼3 dB, but were significantly higher for the 1-kHz tones (*P* < 0.05, *post-hoc* test; Fig. 4B). Indeed, the magnitude of the effect reported here is necessarily conservative as we were initially unable to obtain thresholds using 1-kHz tones for any of the animals tested.

Because acoustical measurements in ferrets have shown that, at low sound frequencies (≤ 3 kHz), ILDs are typically very small and show little variation with sound-source location (Carlile, 1990), the poor low-frequency ILD sensitivity observed here may reflect an inherent limitation of the ferret auditory system. On the other hand, ferrets might be unable to lateralize 1-kHz ILDs simply through inexperience of using different values of this cue under normal hearing conditions. To determine whether this is indeed the case, we trained our ferrets for a prolonged period of time on a 1-kHz ILD lateralization task. To maximize the effects of training, we wanted to minimize the number of ILD values that were either extremely difficult or extremely easy to lateralize. To the extent that performance changes substantially over time, this is not practicable without altering the ILD values presented. We therefore adopted an adaptive method for measuring ILD thresholds (Levitt, 1971), so that we could use the same testing procedure throughout the entire training period.

**Fig. 5:**
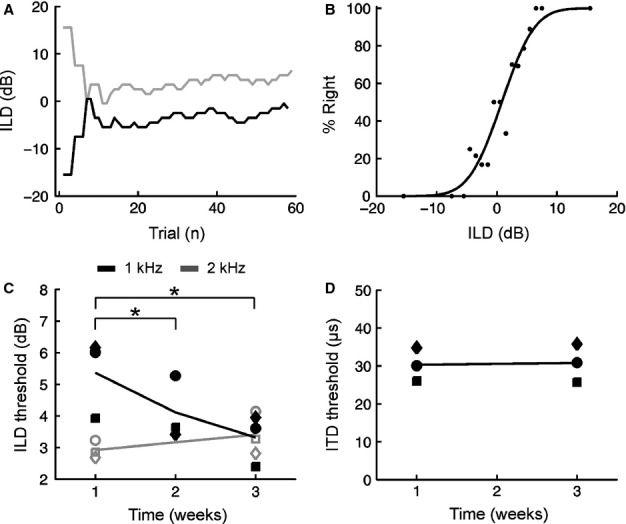
Effect of prolonged training on ILD thresholds using 1-kHz tones. (A) Data obtained from an individual session using an adaptive staircase procedure. Two staircases were randomly interleaved and used to target different points on the psychometric function (29% right, black; 71% right, grey). The ILD values for each staircase are plotted as a function of trial number. (B) Data from A but re-plotted as a psychometric function so that the percentage of trials on which a subject responded to the right is shown as a function of ILD (black markers). Best fit line obtained using probit analysis is shown in black, with thresholds calculated as before. (C) ILD thresholds are plotted as a function of the length of training received. Filled black markers show ILD thresholds obtained from individual subjects using 1-kHz tones, with the corresponding mean across subjects shown by the black line. Unfilled grey markers show 2-kHz ILD thresholds obtained from the same subjects during the first and last weeks of training with 1-kHz tones. (D) ITD thresholds obtained using 1-kHz tones from the same subjects during the first and last weeks of ILD training. Plotting conventions are identical to C. Asterisks denote significant differences (*P* < 0.05, corrected for multiple comparisons).

Using this technique, the ILD values presented were determined by the responses of the subject. Two adaptive staircases were randomly interleaved and used to target two different points on the psychometric function (Fig. 5A). This ensured that ILD cues converged on values that the subjects responded to reliably but imperfectly (i.e. for each staircase, subjects gave the same response on ∼ 71% of trials). For each session, these data were used to construct psychometric functions (Fig. 5B), from which ILD thresholds were subsequently calculated.

Over the course of 3 weeks, during which each animal completed approximately 3000 trials, ILD thresholds gradually improved for 1-kHz tones (*F*_2,85_ = 10.71, *P* = 0.0001; mixed-effects anova; Fig. 5C). This did not reflect a generalized improvement in ILD sensitivity, as occasional testing of ILD thresholds using 2-kHz tones did not show any improvement at this frequency over the same period (*F*_1,17_ = 0.68, *P* = 0.42; mixed-effects anova). The ILD thresholds obtained using 1-kHz tones in the final week of training had improved so much that they were no longer significantly different from those obtained using 2-kHz tones (*F*_1,36_ = 0.01, *P* = 0.91; mixed-effects anova; Fig. 5C). The initially poor ILD sensitivity observed using 1-kHz tones is therefore not due to any inherent limitation in ILD processing at this frequency, but may instead arise from these values typically being of limited behavioural importance, a situation that can be altered with appropriate training. Improvements in ILD thresholds at 1 kHz were also unaccompanied by any obvious improvement in ITD thresholds for the same stimuli (*F*_1,15_ = 0.52, *P* = 0.48; mixed-effects anova; Fig. 5D), which remained relatively constant over time. Together, these results indicate that training-induced improvements in spatial processing were specific to both the trained binaural cue and the sound frequency.

## Discussion

We set out to determine the frequency dependence of binaural cue sensitivity in the ferret, a species that has been widely used for investigating both fundamental (Dahmen *et al*., 2010) and clinically relevant (King *et al*., 2000; Hartley *et al*., 2010; Keating *et al*., 2013a) aspects of spatial hearing. Previous work using broadband noise as a stimulus has shown that the sensitivity of ferrets to ITDs and ILDs is comparable with that of other species, including humans (Keating *et al*., 2013b). Here, we extend this comparison with human hearing by showing that the duplex theory of sound localization, in which these binaural cues operate over different frequency ranges, also applies to ferrets. By combining the frequency specificity of pure tones with the cue specificity afforded by presenting stimuli over earphones, we were also able to investigate whether the usefulness of binaural cues is determined primarily by acoustical or neurophysiological factors.

Although the duplex theory of sound localization (Strutt, 1907) has played a central role in our understanding of how humans localize sounds in the horizontal plane, the spatial hearing of some species deviates considerably from the predictions of the duplex theory (Heffner & Heffner, 2003; Takahashi, 2010; Wesolek *et al*., 2010). Whilst valuable insights into auditory processing can still be gained from these species, the implications for humans become less clear when such differences exist (Heffner & Heffner, 2003). By showing that the duplex theory applies to the ferret, our results therefore confirm the ferret as an excellent experimental model for understanding spatial hearing in humans.

In the case of ITD cues, we found that sensitivity declined as the frequency of the stimulus was reduced, a finding that has been reported in humans as well as other species (Zwislocki & Feldman, 1956; Houben & Gourevitch, 1979; Fitzpatrick & Kuwada, 2001; Scott *et al*., 2007; Brughera *et al*., 2013; Welch & Dent, 2013). In keeping with previous work, we found that this decline could be reasonably well accounted for by assuming that these cues are encoded via frequency-invariant sensitivity to IPDs (Houben & Gourevitch, 1979; Scott *et al*., 2007). In highlighting the possible importance of IPD at a behavioural level, our results are consistent with electrophysiological evidence for the representation of IPD in the auditory system, both at the single neuron (Yin & Kuwada, 1983; Spitzer & Semple, 1995) and at the population level (McAlpine *et al*., 2001).

Nevertheless, in common with previous neurophysiological (Fitzpatrick & Kuwada, 2001) and behavioural (Brughera *et al*., 2013) results, the decline that we observed in ITD sensitivity below 1 kHz was slightly less than that expected by a mechanism that is sensitive to a fixed IPD. Our data therefore lie somewhere between the predictions associated with a mechanism based on frequency-independent IPD sensitivity and one in which neurons are tuned to specific ITDs, independent of the stimulus frequency. This is perhaps what would be expected if the brain were to contain a hybrid representation of timing cues in which both IPDs and ITDs are represented. Consistent with this view, there is evidence to suggest that the relative importance of IPD and ITD may vary at different levels of the auditory pathway through changes in the way these cues are processed (Fitzpatrick & Kuwada, 2001; Vonderschen & Wagner, 2012), which could reduce the frequency-dependence of ITD processing at a behavioural level (Zhang & Hartmann, 2006).

As is the case with humans (Zwislocki & Feldman, 1956; Brughera *et al*., 2013), macaque monkeys (Scott *et al*., 2007), cats (Wakeford & Robinson, 1974) and budgerigars (Welch & Dent, 2013), in ferrets, ITD sensitivity declined at frequencies above 1 kHz. Higher thresholds were obtained at 2 kHz and none of the animals was able to lateralize 3- or 4-kHz tones on the basis of ITD. Although the upper limit for pure tone ITD sensitivity is ∼ 1.4 kHz in humans (Zwislocki & Feldman, 1956; Brughera *et al*., 2013), the corresponding limit in cats and macaques is at least 2 kHz (Wakeford & Robinson, 1974; Scott *et al*., 2007). Whilst differences between species in the upper limit of phase locking may contribute to this, these results can also be predicted on the basis of phase ambiguity. This is because differences in head size will determine the frequencies at which phase ambiguity is experienced. For example, whereas humans are expected to experience phase ambiguity at approximately 1.4 kHz, the same stimuli should be unambiguous for macaques (Fig. 3D).

Importantly, previous work has shown that macaques can localize 2-kHz stimuli in the free field, but only if these stimuli are presented close to the midline (Brown *et al*., 1978). This intriguing result is difficult to explain on the basis of phase locking, but is a natural consequence of the maximum unambiguous ITD illustrated in Fig. 3D, which shows that ITDs of up to ∼ 100 μs only are spatially unambiguous at this frequency. It is therefore likely that phase ambiguity contributes to the upper limit of tonal ITD sensitivity in at least some species. By taking into account its relatively small head size, however, we were able to show that phase ambiguity is unlikely to affect ITD sensitivity in the ferret at 2 kHz, a trait which it probably shares with other animals that have small heads (Heffner & Heffner, 1987; Welch & Dent, 2013).

Instead, our results are best explained by recently published data obtained from the ferret auditory nerve, which show a reduction in phase locking between 1 and 3 kHz (Sumner & Palmer, 2012). In the ferret, the upper limit of tonal ITD sensitivity is therefore likely to be determined primarily by the neurophysiological characteristics of the auditory nerve. Indeed, it is possible that this species does not experience phase ambiguity at all, as such ambiguity would only be expected to occur for frequencies at which the ferret is insensitive to the phase of the acoustic input. The ferret may therefore not require specific neural mechanisms for resolving phase ambiguity (Peña & Konishi, 2000). In this respect, species such as the ferret, which possess a relatively small head size and comparatively low phase-locking limit, may differ from species that have larger heads and/or which show phase-locked responses up to higher frequencies.

Consistent with this view, it has been proposed that head size may affect the optimal coding strategy for representing ITDs (Harper and McAlpine, 2004). In species with a very small head, for example, theoretical considerations predict two distinct subpopulations of neurons tuned to ITDs outside the physiological ITD range. Species with a much larger head, however, are expected to show a homogeneous distribution of neurons tuned to ITDs that lie within the physiological range. For many species, Harper and McAlpine (2004) predicted a transition between these two coding strategies as sound frequency is increased. Interestingly, the transition point between these two coding strategies coincides with the frequency at which phase ambiguity begins to occur for peripheral locations. It is therefore possible that phase ambiguity might contribute to this change in coding strategy.

Because head size changes as an animal grows, the impact of phase ambiguity is likely to be developmentally regulated. In addition to the maturation of phase locking (Kettner *et al*., 1985) and binaural circuits (Seidl & Grothe, 2005), the impact of increasing head size on phase ambiguity therefore also needs to be considered, at least in some species, when attempting to account for the emergence of spatial hearing abilities for low-frequency sounds during the course of development.

In budgerigars (Welch & Dent, 2013), cats (Wakeford & Robinson, 1974), macaques (Houben & Gourevitch, 1979; Scott *et al*., 2007) and humans (Mills, 1960; Yost & Dye, 1988), it has been shown that ILD sensitivity is relatively constant with respect to sound frequency over a frequency range similar to that tested in this study. Nevertheless, because of the way sounds interact with the head and ears, ILDs will only be generated by distal free-field sources at relatively high frequencies. In these species, the usefulness of ILD cues therefore appears to be limited by acoustical factors alone.

In contrast to other species, however, ferrets were very poor at lateralizing 1-kHz ILDs when they were first tested, showing much higher thresholds at 1 kHz than those obtained at higher frequencies. One possibility is that the initially poor ILD sensitivity that we observed at 1 kHz, a frequency at which ferrets are particularly sensitive to ITDs, might reflect the effects of prior experience concerning the relative usefulness of different spatial cues. Our ferrets were trained on the procedural aspects of the lateralization task using broadband noise, and were only subsequently tested with pure tones. Because of the availability of ITDs at this frequency, the animals may have been relatively inexperienced in using 1-kHz ILDs for determining azimuth (not necessarily distance, Jones *et al*., 2013) when their thresholds were initially measured. This approach contrasts with previous animal studies that have tended to provide subjects with experience of test stimuli during preliminary training, potentially improving their perceptual capabilities prior to the measurement of thresholds (Wakeford & Robinson, 1974; Houben & Gourevitch, 1979; Scott *et al*., 2007).

Consistent with this possibility, we found that ILD sensitivity at 1 kHz improved considerably when ferrets were given prolonged training, indicating that the relatively high thresholds initially obtained at this frequency do not reflect an inherent limitation of the auditory system in this species. This improvement was not due to a generalized change in ILD sensitivity, as there was no concomitant improvement in ILD sensitivity for 2-kHz tones. Given appropriate training, the frequency dependence of ILD sensitivity can therefore be reduced in the ferret. This means that the precise transition point between ITD and ILD sensitivity may be experience-dependent, a finding that has important implications for neurophysiological measurements of binaural cue sensitivity.

Perhaps the most important implication of this experience-dependent plasticity, however, is that mammals can learn to use spatial cues that are typically unavailable under normal acoustical conditions, and can learn to do so even during adulthood. In showing that such plasticity can occur in the ferret, this study paves the way for investigating the underlying neural basis. In the case of subjects with normal hearing, it might be argued that this type of plasticity is of little benefit. However, if individuals were provided with an assisted-hearing device that converts spatial information into low-frequency ILDs, then learning to use these unnatural cues could provide a valuable method for improving spatial hearing. This is likely to be particularly beneficial for individuals who suffer from hearing loss. Thus, whilst the plasticity we report here provides basic insight into adult plasticity and the mechanisms underlying spatial hearing, the implications of these findings may also help guide rehabilitation strategies for individuals whose hearing has been compromised.

## Abbreviations

ABL: average binaural level
ILD: interaural level difference
IPD: interaural phase difference
ITD: interaural time difference
